# A double paradox: end-stage renal disease—high thromboembolic and bleeding risk management using a device with risks of thromboembolism and bleeding

**DOI:** 10.1093/europace/euaf238

**Published:** 2025-09-23

**Authors:** Tatjana Potpara, A John Camm

**Affiliations:** Medical Faculty, University of Belgrade, Belgrade, Serbia; Cardiology Clinic, University Clinical Centre of Serbia, Belgrade, Serbia; Genetic and Cardiovascular Sciences Institute, Cardiology Academic Group, City St. George’s University of London, Cranmer Terrace, London SW17 0RE, UK

**This editorial refers to ‘Left atrial appendage occlusion in patients with end-stage renal disease: an individual patient-level meta-analysis’ by J.F. Rodriguez-Riascos *et al.*, https://doi.org/10.1093/europace/euaf198.**

*How wonderful that we have met with a paradox. Now we have some hope of making progress.*

**—**Niels Bohr

We have little certainty of the best way to manage patients with the combination of atrial fibrillation, a high stroke risk, and renal failure, but what do we know?

Although oral anticoagulation (OAC) effectively reduces thromboembolic stroke in patients with atrial fibrillation (AF) at high risk of stroke, it is also associated with an increase in long-term bleeding. However, net clinical benefit generally favours anticoagulation, especially when using direct oral anticoagulants (DOAC).^1^ Patients greatly prefer suffering from a major bleed, which is usually remediable, compared with sustaining a stroke, the adverse consequences of which may be permanent.^2^ This net benefit extends to patient-reported outcomes and quality of life. However, there are groups of patients where the increased threat of bleeding or bleeding history precludes the use of anticoagulation or reverses the risk-benefit to such an extent that doctors, carers, and patients prefer to forgo the antithrombotic protection conferred by OAC. Such groups include AF patients with bleeding diatheses, repeated uncorrectable gastrointestinal bleeding, intracranial bleeding, end-stage renal disease (ESRD), etc.^3^ The latter group poses a particular problem because renal failure promotes bleeding and is also associated with increased thrombosis and embolism.^4^

In practice, renal physicians may opt to recommend vitamin K antagonist (VKA) therapy, which is easily monitored, DOAC, which have far fewer drug–drug or food–drug interactions, or nothing for ESRD patients with AF, particularly those undergoing haemodialysis. The increased thromboembolism and bleeding associated with such patients have been well documented, but the value of OAC, even with VKAs, has not been shown consistently to lead to better outcomes in these patients.^5^ In the large pre-approval DOAC vs. VKA randomized controlled trials, patients with mild to moderate renal impairment showed results similar to those without renal impairment that is a reduction in stroke but a small increase in major bleeding.^6^

Patients with severe renal impairment were excluded from these studies. Given this, attempts were made in this population to derive sufficient data from observational studies to warrant prospective randomized trials. Comparisons were made against historical control data from patients not anticoagulated or patients treated contemporaneously with VKAs.^7^ Given the pace of medical progress, positive comparisons against historical controls were treated with considerable scepticism, but comparisons, for example, of apixaban^8^ or rivaroxaban^9^ against warfarin, were sufficiently compelling to encourage the Food and Drug Administration to offer specific dosing advice for the use of these DOAC in AF patients with end-stage renal failure. Attempts have been made to prospectively compare randomized or non-randomized groups of DOAC-treated against VKA-treated AF patients. The randomized studies have generally failed because of poor recruitment and little prospect of demonstrating conclusive results,^10^ whereas the non-randomized comparisons have shown that rivaroxaban and apixaban, for example, is better than VKA treatment, but the non-randomized nature of group assignment does not allow any firm conclusion.

**Figure 1:**
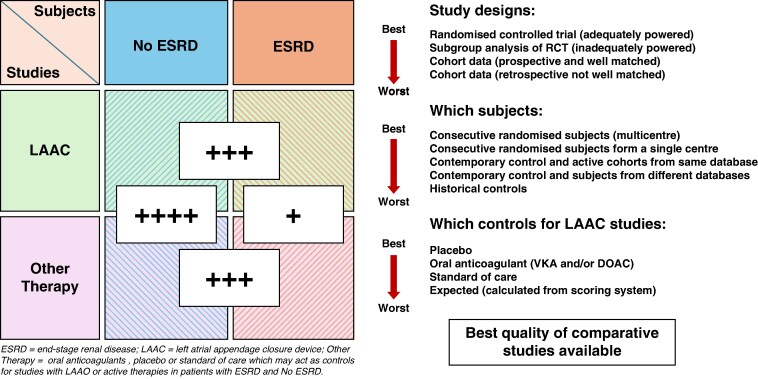
Range and quality of studies available for the assessment of left atrial appendage therapy in patients with end-stage renal disease.

Given that the data so far accumulated is not sufficient to convincingly demonstrate any value of anticoagulation in AF patients with ESRD but does raise considerable suspicion that such treatment is associated with an increased bleeding hazard, it is legitimate to consider whether a completely different approach to thromboprophylaxis might offer a better solution. During the last two decades, closing the left atrial appendage, the site at which 90% of left atrial thrombus forms, with a device that can be inserted via a catheter and expanded to occlude the appendage [left atrial appendage closure (LAAC) device] and prevent the escape of thrombus, has been shown to reduce stroke similarly to VKAs and reduce long-term major bleeding complications.^11,12^ Major studies are now underway to specifically compare direct oral anticoagulant treatment with LAAC, and some results are expected shortly (*Figure 1*).

These large studies do not include patients for whom OAC is contraindicated. Currently, we have only observational studies, comparing retrospective or prospective outcomes in patients with AF and ESRD compared to expected rates based on bleeding and stroke scores or with anticoagulants or more often standards of care that do not involve anticoagulation. The study reported by Genovesi *et al*.^13^ is of substantial interest. This registry study compared 106 AF dialysis patients treated with LACC implant, 114 treated with warfarin, and 148 with no thromboembolic protection. After adjustment for known confounders, the bleeding risk for LAAC was significantly lower than for warfarin, and the stroke rate with LAAC was significantly lower than treatment with warfarin or no antithrombotic and lower than expected according to the CHA2DS2-VASc score. In these studies, comparison groups are often closely matched or adjusted to minimize bias, but some bias inevitably remains. Given this concern, however, these studies have usually shown a very significant reduction in these outcome events when LAAC is employed.

Other observational studies compare the use of LAAC in patients with and without ESRD.^14^ These studies generally confirm that in-hospital mortality, stroke, and major bleeding are higher, but LAAC procedural complication rates are no greater in patients with ESRD than in those without renal disease. Very importantly, stroke and long-term major bleeding following LAAC are similar in both groups. One large study of this nature, which utilized retrospectively collected data from the US Renal Data System registry, was recently reported by Dhar *et al*.^15^ Of 2344 patients with kidney failure and a recent diagnosis of AF, 293 were treated with LAAC. Propensity matching was used to reduce confounders, after which the study showed improved survival and reduced bleeding risk associated with LAAC. Non-fatal strokes were similar in both groups. The very large study reported by Jamal et al., which compared 2016–17 hospitalizations in 16 505 patients receiving an LAAC device, of which 3245 had chronic kidney disease and ESRD, demonstrated that procedure-related complications and in-hospital mortality were no different in those with and without kidney disease.

In this issue, Rodriguez-Riascos *et al*.^16^ present a meta-analysis, which includes 24 127 patients, including 1047 with ESRD. It explored the value of LAAC in patients with and without ESRD. Unlike some previous studies, the results showed that procedural complications were twice as common in patients with ESRD. Although there was no significant difference in thromboembolism rates during follow-up between the groups, not surprisingly, the incidence of major bleeding was higher among patients with ESRD. Major implant complications, such as peri-device leak and device-related thrombus, were no greater in those with ESRD.

Against this background, how should we approach thromboprophylaxis in patients with ESRD? There is clearly more intrinsic disease-based thromboembolic and bleeding hazard in ESRD patients, but this difference may no longer apparent after LAAC implant. Some long-term complications, such as major bleeding, are greater in those with than in those without serious kidney disease, but LAAC is a relatively safe procedure even in patients with ESRD.^15^ Overall, there is no serious drawback to LAAC in ESRD patients. When OAC appears unwarranted or unsafe, LAAC therapy is a clear therapeutic option. At present, professional society guidelines do not identify these patients specifically as candidates for LAAC therapy, but the general wording of recommendations (LAAC ‘… may be considered in patients with AF and contraindications for long-term anticoagulation …’ European Society of Cardiology 2024^17^ or ‘In patients with moderate to high risk of stroke and a contraindication to long-term anticoagulation … “LAAC …” is reasonable’ ACC/AHA/ACCP/HRS)^18^ gives limited recognition to this option.

We await with great interest the large studies of LAAC vs. DOAC therapy in high stroke risk patients with AF, but it is doubtful that many, if any, patients with ESRD will be recruited into these studies. Smaller studies that investigate groups at particular or unknown risk from anticoagulant therapy, amongst which are patients with ESRD, may provide more definitive data. COMPARE-LAAO (NCT04676880) has recruited patients with documented AF and a CHA2DS2-VASc risk score ≥ 2 who are unsuitable for long-term OAC.^19^ Patients with severe renal disease are not specifically excluded from this trial, which will randomize LAAC vs. OAC (2:1), and the primary endpoint will be the time to first occurrence of the composite of stroke, transient ischaemic attack, and systemic embolism. CLOSURE-AF (NCT03463317), which randomizes patients at high risk of bleeding, unfortunately does not include patients with ESRD.^20^ However, the Left Atrial Appendage Closure in Patients With Non-valvular Atrial Fibrillation and End-stage Chronic KIDNEY Disease (LAA-KIDNEY) trial (NCT05204212) is randomizing patients with documented non-valvular AF (paroxysmal, persistent, or permanent), a CHA2DS2-VASc risk score ≥ 2, and ESRD (KDOQI stage 5, eGFR < 15 mL/min/1.73 m^2^, with or without haemodialysis) to LAAC or best medical care. The primary outcome is a net benefit composite of time to first stroke, systemic embolus, cardiovascular death, or major bleeding. It is not anticipated to report until 2028. However, for the time being, it seems increasingly probable that LAAC may be assumed to be a reasonable option for the management of patients with AF-related stroke risk and renal failure.

*Medicine is a science of uncertainty and an art of probability.*

—William Osler
